# Forgotten ureteral stents: a systematic review of literature

**DOI:** 10.1186/s12894-024-01440-9

**Published:** 2024-03-05

**Authors:** Xiaochuan Wang, Zhengguo Ji, Peiqian Yang, Jun Li, Ye Tian

**Affiliations:** Department of Urology, Beijing Friendship Hospital, Capital Medical University, No. 95, Yongan Road, Xicheng District, Beijing, China

**Keywords:** Forgotten ureteral stent, Systematic review, Ureteral stent complication, Endoscopic stent removal, Prevention strategy

## Abstract

**Background:**

The forgotten ureteral stents (FUS) is one of the late complications of stent placement. This systematic review summarized different aspects of FUS and focused on the problems and solutions related to FUS.

**Methods:**

This systematic review was conducted in accordance with the Preferred Reporting Items for Systematic Reviews and Meta-analyses (PRISMA) statement. PubMed® and Embase® were searched from inception until October 1st, 2022. Eligible studies were those defining FUS as a stent unintentionally left in situ longer than at least 2 months.

**Results:**

Total 147 studies with 1292 patients were finally included. The mean indwelling time of FUS was 33.5 months (range from 3 months to 32 years). The most common initial cause for stent placement was adjunct treatment to urolithiasis (79.2%). The major forgetting reasons were patient-related (83.9%), which included poor compliance, lapse in memory, and misconceptions about the necessity of timely removal. Primary presenting complaints were flank pain (37.3%), lower urinary tract symptoms (33.3%), and hematuria (22.8%). Encrustation (80.8%) and urinary tract infections (40.2%) were the most common complications detected in patients with FUS. Computed tomography evolving as a preferred imaging test (76.1%) was indispensable for evaluating encrustation, migration, fracture and other complicated situations in patients with FUS. Besides, evaluation of kidney function and infection status was also of great importance. Multiple and multimodal procedures (59.0%) were often necessitated to achieve the stent-free status, and were mostly endoscopic procedures. Cystoscope was most commonly used (64.8%). Retrograde ureteroscopy (43.4%) and antegrade stent removal (31.6%) were often used when dealing with more complicated situations. Extracorporeal shockwave lithotripsy (30.4%) was often used as adjunctive to other endoscopic procedures, but it sometimes failed. The decision regarding the choice of treatment is based on the volume and site of encrustation, the direction of migration, the site of fracture, kidney function and other urinary comorbidities.

**Conclusions:**

FUS not only pose hazard to patients’ health, but also impose a huge economic burden on medical care. Thorough preoperative evaluation is fundamental to developing the treatment strategy. The management of FUS should be individualized using different treatment modalities with their advantages to minimize patients’ morbidities. Prevention is better than cure. Strengthening health education and setting a tracking program are of great importance to the prevention of FUS.

**Supplementary Information:**

The online version contains supplementary material available at 10.1186/s12894-024-01440-9.

## Background

Ureteral stents, especially double-J stents are currently one of the most widely used surgical tools in the field of urology. The first ureteral stent for long-term retention was used by Zimskind and associates in 1967 [1]. The pigtail shape design described by Finney in 1978 [2] and a variety of innovations and developments [3] laid the foundation for modern stents. Stent insertion is the most efficient way to relieve ureteral obstruction, and it was indispensable to many surgical interventions to promote ureteral healing and to prevent complications. However, some stent-related problems may develop, such as irritative symptoms, urinary tract infections (UTI), encrustation, migration and fracture [4].

Technological advances in stent design, constitutive materials and surface coating allow patients to tolerate stents more easily, and this may cause a decrease in patient compliance for stent removal [5]. Studies have found that stents were forgotten in up to 0.9–12.0% of patients [6]. Ureteral stents as foreign bodies should be removed or replaced after they have served their purposes before the intended maximal stent life (MSL). The forgotten ureteral stent (FUS) with long-term use tends to migrate, encrust and fracture, and can lead to severe sepsis, renal failure and even a life- threatening situation [7, 8]. Therefore, the management of FUS presents a considerable surgical challenge for urologists and an increased morbidity to patients, usually requiring multimodal treatments to render stent-free. In addition to potential legal consequences [9], the cost of removing complicated stents was estimated to be 1.8- to 21-fold higher than a regular stent, and financial burden of FUS management increased in parallel with the duration of the stent retention [10].

This systematic review provides an overview of FUS and pools data regarding patients’ demographics, diagnosis, management and prevention to improve urologist understanding of FUS management.

## Methods

### Search strategy

A comprehensive search for eligible studies was conducted using PubMed® and Embase® from inception until October 1st, 2022. The Preferred Reporting Items for Systematic Reviews and Meta-Analyses (PRISMA) 2020 statement was followed in this review [11]. The search was restricted to English using search terms included ‘forgotten ureteral/ureteric stent’, ‘retained ureteral/ureteric stent’, ‘overlooked ureteral/ureteric stent’, ‘missed ureteral/ureteric stent’ or ‘neglected ureteral/ureteric stent’ in previous literatures.

### Study selection

**Fig. 1:**
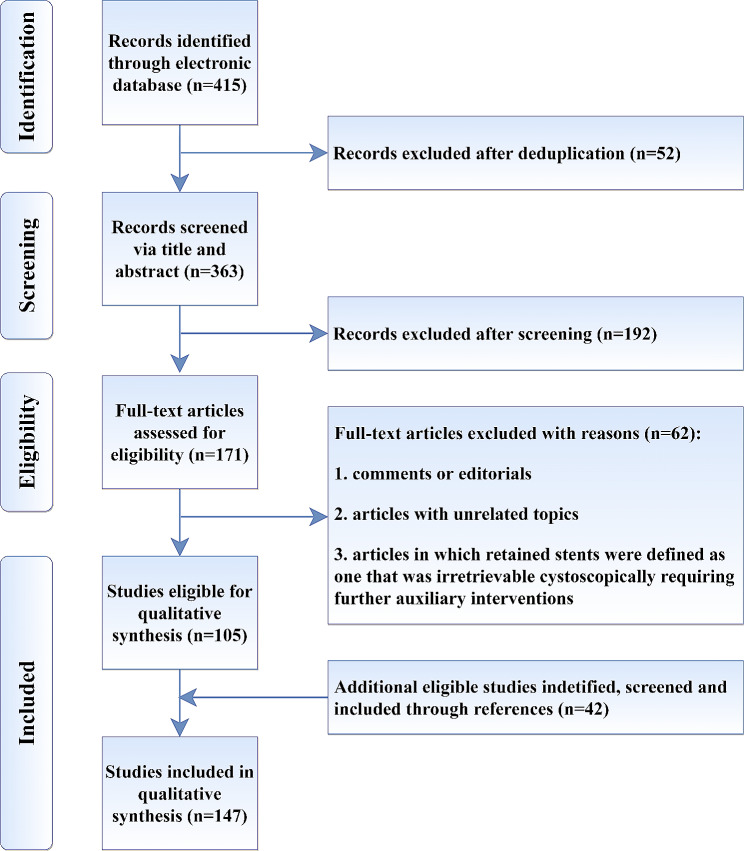
PRISMA flowchart of the included studies

After deduplication of retrieval records, the abstracts were independently screened for eligibility by two authors (X.W. and Z.J.), followed by independent retrieval and scrutiny of full-text articles. Any discrepancies were resolved by discussion or by consulting a third author (P.Y.). Studies defining FUS as a stent unintentionally left in situ longer than at least 2 months were included. The PRISMA flow chart with details of exclusion criterion were shown in Fig. 1.

### Data analysis

We reported FUS-related data, such as epidemiology, aetiology, diagnosis, management and prevention. The numerical data obtained from available studies were synthesized and calculated. The mean or median (with standard deviation or range, if available) was reported for continuous variables, while a constituent ratio was reported for each category of categorical variables. All statistical tests were performed by SPSS version 24.0 (IBM, Armonk, NY, USA).

## Results

### Description of the included studies

Four hundred and fifteen records were initially retrieved from electronic databases. After deduplication, 363 abstracts were screened and 171 full-text articles were reviewed. The exclusion criteria are listed in Fig. 1. Finally, 147 articles were included with 1292 patients. A narrative synthesis rather than a quantified meta-analysis of data was performed. All included 147 articles are shown in Supplementary Material.

### Study characteristics

All 147 articles were published from 1985 to 2022 with a rising tendency in publication numbers by year. Seventy-eight (53.1%) articles were published in recent past 10 years. There are 109 case reports including 130 patients with 141 stents and 38 case series (> 3 cases) including 1162 patients with 1182 stents. The most commonly used terms for FUS are ‘forgotten’. The criteria for indwelling time of FUS has been defined in previous studies (case series) as a variable period more than ranging from 3 to 12 months. (Table 1)

**Table 1:** The definition of FUS used in the included studies

Definition of FUS	% (n)
Terms used (*n* = 147)	
Forgotten	66.7 (98)
Lost to follow-up	12.2 (18)
Retained	8.2 (12)
Neglected	6.1 (9)
Missed	3.4 (5)
Long indwelling	2.7 (4)
Overlooked	0.7 (1)
Case series: criteria* for indwelling time of FUS (*n* = 38)	
>3 months	26.3 (10)
>6 months	47.4 (18)
>12 months	26.3 (10)
Case reports: indwelling time of FUS (*n* = 130)	
3–6 months	3.8 (5)
6–12 months	3.1 (4)
1–2 years	14.5 (19)
2–5 years	27.5 (36)
5–10 years	29.0 (38)
10–20 years	16.0 (21)
>20 years	5.3 (7)

FUS, forgotten ureteral stent

* If no criterion was defined, then the minimum indwelling time of FUS which was mentioned in the article was used as the cut-off point.

### Patient demographics, initial causes for placement and forgetting reasons

The mean age of included patients was 41.5 years with age range from 2 to 92 years. Pediatric and adolescent patients accounted for 7.3%. Male to female ratio was 1.85. The initial causes for stent placement in patients with FUS fall into 4 categories (Table 2): Stent placement as an adjunct to the stone treatment was the most common reason (79.2%) for stent placement.

**Table 2:** Initial causes for the placement of forgotten ureteral stents

Initial causes (*n* = 1182)	% (n)
Adjunct to stone treatments	79.2 (936)
Endourological procedures	34.4 (407)
Pyelolithotomy and ureterolithotomy	11.1 (131)
Extracorporeal shockwave lithotripsy	8.1 (96)
Relieve obstruction for emergency	2.8 (33)
Unknown stone treatment (Not reported)	22.8 (269)
Adjunct to other surgeries	12.0 (142)
Pyeloplasty	4.6 (54)
Ureter reimplantation	1.9 (22)
Renal transplantation	1.7 (20)
Urinary diversion (ileal conduit)	0.6 (7)
Unknown urological surgeries (Not reported)	1.9 (22)
Nonurological surgeries (for identification of ureters)	1.4 (17)
To relieve obstruction for long-term stenting	6.3 (75)
Benign diseases	2.9 (34)
Malignant diseases	2.3 (27)
Pregnancy	1.2 (14)
To promote recovery for injuries	0.8 (10)
Abdominal trauma	0.6 (7)
Radiation and iatrogenic injury	0.3 (3)
Not reported	0.5 (6)

The major forgetting reasons were patient-related (83.9%), which included poor compliance, lapse in memory, and misconceptions about the necessity of timely removal. The second most common reasons were physician-related (24.7%) and attributed to inadequate counseling. Besides the above, objective factors (4.4%) such as individual financial problem, necessity to treat other diseases, low education status and social instability also led to the delay for stent removal.

### Clinical manifestations and diagnostic tests

The mean indwelling time was 33.5 months (range from 3 months to 32 years). A 59-year-old woman suffered from heavily encrusted bilateral stents for 32 years, which was the longest indwelling time in previous literatures [12]. The “32-year-old” stents were inserted for a prophylactic use in a hysterectomy. The distribution of the indwelling time of FUS in 130 case reports is detailed in Table 1. Bilateral FUS and FUS in solitary kidneys are both uncommon (2.4% and 2.5% respectively).

The most common primary presenting complaints of FUS were flank pain (37.3%), lower urinary tract symptoms (33.3%) and hematuria (22.8%), which are so-called stent-related symptoms. Encrustation (80.8%) and UTIs (40.2%) were the most common complications. More details are shown in Table 3.

**Table 3:** Clinical manifestations of forgotten ureteral stents

Primary presenting complaints (*n* = 880)	% (n)
Flank pain*	37.3 (328)
LUTS	33.3 (293)
Haematuria	22.8 (201)
Dysuria	18.0 (158)
Fever or systemic infections	19.2 (169)
Asymptomatic	10.8 (95)
Uraemic symptoms	4.2 (37)
Stenturia**	1.0 (9)
Complications (total n)	
Encrustation (1303)	80.8 (1053)
UTIs, positive urine culture (880)	40.2 (354)
Elevated sCr or impaired eGFR (392)	25.8 (101)
Spontaneous fracture (949)	16.1 (153)
Migration (951)	7.2 (68)

*few patients presented with suprapubic pain; ** passage of small stent fragments from urine;

eGFR, estimated glomerular filtration rate; LUTS, lower urinary tract symptoms; sCr, serum creatinine; UTI, urinary tract infection

KUB (kidney-ureter-bladder) radiography (96.8%), non-contrast computed tomography (NCCT) (76.1%) and KUB ultrasonography (45.2%) were commonly used for imaging evaluation. Elevated serum creatinine was detected in 24.8% of patients, and the rate increased to 62.5% in patients with bilateral FUS. Renal scintigraphy was preferred in 35.5% of cases to quantitatively estimate the split renal function of affected kidneys.

### Management

Multiple sessions or modalities were necessitated to render stent-free status for most patients (59.0%). Simple cystoscopic stent removal (SCSR) with or without endoscopic cystolithotripsy (EnCL) was the most commonly used procedures (64.8%) for FUS removal and associated stones. Retrograde ureteroscopic stent removal with or without intracorporeal lithotripsy (43.4%) and antegrade stent removal with or without percutaneous nephrolithotomy (PCNL) (31.6%) were often used when SCSRs failed. Extracorporeal shockwave lithotripsy (ESWL) (30.4%) was often used followed by other endoscopic procedures. Pretreatment with percutaneous nephrostomy (3.6%) and antibiotics may be needed for complicated UTIs. Laparoscopy and open surgeries such as pyelolithotomy, ureterolithotomy, cystolithotomy, ureteral reimplantation, pyeloureteroplasty and even nephrectomy was performed to deal with more complicated situations (6.7%) which included a huge stone burden, a non-functioning kidney and comorbid stricture or malformation. Postoperative complications were uncommon, however, not a few complications were severe or even lethal. More details are shown in Table 4.

**Table 4:** Management of forgotten ureteral stents

Management (*n* = 1322)	% (n)
Modality	
SCSR with or without EnCL	64.8 (857)
Retrograde USR with or without intracorporeal lithotripsy	43.4 (575)
Antegrade stent removal with or without PCNL	31.6 (418)
Endoscopic stent removal with ESWL	30.4 (402)
Laparoscopy and open surgery	6.7 (87)
Multiple sessions or modalities	59.0 (780)
Preoperative percutaneous nephrostomy	3.6 (48)
Postoperative placement of new stents (*n* = 434)	40.1 (174)
Stent-free rate	99.0 (1310)
Postoperative complication rate (*n* = 747)	
Fever and sepsis	9.5
Haematuria	2.4
Ureteral injury	2.0
Death	0.6

EnCL, endoscopic cystolithotripsy; ESWL, extracorporeal shockwave lithotripsy; PCNL, percutaneous nephrolithotomy SCSR, simple cystoscopic stent removal; USR, ureteroscopic stent removal;

## Discussion

### Study characteristics

Technically, FUS is defined as a stent of which the indwelling time exceeds the MSL. In a broad sense, FUS is also defined as a stent unintentionally left in situ despite the physician’s recommendation. MSL was recommended for different products provided by various companies [13]. However, in previous literatures, the term “MSL” was not always applied and a strict definition for “forgotten” did not exist. We recommended to use the term “forgotten”, and report the MSL or the intended indwelling time recommended by the physician.

### Forgetting reasons and prevention strategies

The best treatment for FUS is prevention. Meticulous patient education which increases patient’s insight into the importance of both stent insertion and its timely removal includes explaining possible complications of indwelling stents, highlighting the importance of drinking enough water and avoiding excessive exercise, minimizing patient-specific lithogenic factors, undergoing appropriate antimicrobial treatment and making an appointment with patients to ensure their timely follow-up.

Despite extensive counseling, up to 10% of patients with retained stents were lost to follow-up and failed to have their stents removed [14]. Therefore, patient education alone could not be solely relied upon, and it is necessitated to set a monitoring program for tracking the patients with long-term indwelling stents. Computerized monitorization programs, stent removal software and reminder short message or e-mail services have been recommended [9].

The exact interval for changing or removing an indwelling ureteral stent was controversial. An optimum interval is usually 2–6 months, but it should be sooner in patients with risk factors, such as stone history, pregnancy, recurrent encrustation and UTIs [15, 16]. Novel stent coatings and degradable stents have also been investigated as a strategy to prevent bacterial adherence, encrustation and subsequent FUS [17, 18].

### Clinical manifestations and diagnostic tests

Encrustation was thought to be the result of ionic deposition on the biofilm, and it usually begins to agglomerate at both ends. Encrustation along the stent in mid-ureter is relatively uncommon and mild [16, 19]. Common risk factors for stent encrustation are long indwelling time, UTIs, chronic renal failure, recurrent or residual stones, lithogenic history, metabolic abnormalities, congenital renal anomalies and ureteral obstruction, of which indwelling time and history of urolithiasis were major contributing factors [17, 19]. The FECal (forgotten, encrusted, calcified) grading system described by Acosta-Miranda et al. [20] and the KUB grading system defined by Arenas et al. [21] are commonly used to evaluate encrusted stents, and are also useful tools to help urologists to make decisions in the management of FUS.

The migration and the spontaneous fracture (fragmentation) were uncommonly seen. Mild or moderate migration manifested as the proximal or distal end migrating into the ureter. Severe migration manifested as the stent totally migrating into the renal pelvis or bladder, and even the proximal or distal end protruding into the retroperitoneum or out of urethra [22, 23]. The reason for migration is primarily due to the short stent for the ureter [24]. An appropriate length of stents should be chosen, and full loops should be kept in both the pelvic and bladder, especially in children. Long indwelling time is the leading risk factor for broken stents. Fracture could also occur during the extraction of FUS; therefore, gentle traction should be used and the integrity of the stent should be examined and confirmed after stent removal [17]. Fracture was thought to be the result of loss of tensile strength, which was due to the hardening and degeneration of stent polymers [25]. The risk of both fracture and encrustation is dependent on the type of stent material. Silicone was found to be less prone to fracture and encrustation than polyurethane [18].

A thorough preoperative imaging evaluation is crucial to decide on the treatment strategy. KUB radiography was performed to preliminarily evaluate the degree and site of encrustation, the associated stone burden and the location information of migration or fracture. KUB ultrasonography was usually used as an ancillary examination, however, it should be the first choice for the pregnant. NCCT could help assess the exact stone burden and the extent of encrustation, which are underestimated by KUB radiography [16, 19, 26]. In addition, since bowel preparation is hard to achieve in children, intensive gas on KUB could mask visualization of the actual stone burden [27]. NCCT is also useful in evaluating comorbidities in urinary system and adjacent organs (such as colon, rectum, and uterus). Therefore, NCCT has become a preferred and indispensable modality in recent years to diagnose FUS, especially the one with severe encrustation and other complicated situations.

Kidney function was mainly focused, especially in patients with a solitary kidney or bilateral severely encrusted stents. It is demonstrated that patients with FUS are at increased risk for loss of renal function [28]. The estimated glomerular filtration rate at diagnosis of FUS was significantly lower than that at the time of stent insertion [29]. UTIs and urosepsis also cause great attention of physicians. Long-term indwelling stents offer an ideal surface for bacterial colonization and biofilm formation. The adherent bacteria which hydrolyze the urea to ammonia increase the urinary pH which leads to precipitation of minerals [30]. Bacteriuria is a strong contributing factor for stent encrustation and stone formation.

FUS was detected incidentally in 10.8% of patients, and more than a few patients carried stents for years and decades until symptoms occurred. It was described that FUS was more common in patients who tolerated stents well than in those who had discomfort [5].

### Management

There are currently no formal guidelines but several treatment algorithms in the management of FUS. Single-session removal is often discouraged, and it is better to stage the procedures to avoid long intraoperative time and resultant complications [18]. With improvements in surgical position [34] and techniques [5], removing fractured or encrusted FUS in a single endourologic session could be achieved with reasonable operating time and acceptable morbidity. At an experienced center, combined endourological procedures can achieve safe and successful management even in the pediatric group [29]. A complex situation often involves the kidney, ureter and bladder, necessitating multimodal endoscopic procedures and even a more invasive surgery that may be performed either simultaneously, sequentially or separately. Each treatment modality has its advantages and disadvantages, and therefore a treatment strategy should be devised individually. The strategy is mainly based on the volume and site of encrustation, the direction of migration, the site of fracture, kidney function and other urinary comorbidities. It is recommended to deal with the distal ends first in order to facilitate subsequent procedures such as ESWL and PCNL [15], and also facilitate placing a ureteral access catheter or a parallel stent [7].

SCSR or semi-rigid ureteroscopes alone could be performed for distal ends with no or minimally encrustation and simply downward migrated FUS. Although severely or circular encrustation completely encasing the distal end could be management by SCSR + EnCL [31], the semi-rigid ureteroscope combined with lithotripsy devices has an advantage in dealing with ureteral part, broken stent pieces left in situ after retrograde traction, and upward migrated FUS in the ureter [14, 16].

It is demonstrated that ESWL cannot be successful alone, and may offer less help in cases with severe encrustation and a large stone burden. However, as a noninvasive treatment, ESWL may increase the potential success of subsequent endourological procedures [32]. Therefore, in cases with failure of retrograde removal, the initial adjunctive use of ESWL (1–3 sessions) on proximal ends may be efficacious, and ESWL is also useful in disintegrate the encrustation on the ureteral part [5, 16].

Proximal stone burden is described as a main determining factor in the management of FUS, and correlated with multiple sessions, multimodal procedures and complications [28, 33]. Antegrade stent removal (with PCNL) alone was performed when FUS was evaluated only having encrusted proximal coil, associate renal stones or upward migration. However, PCNL is usually combined with other retrograde procedures, or performed when ESWL failed [19]. Flexible ureteroscopy is used in some selected cases having uncoiled proximal ends with encrustation, and it is also useful to manage upper ureteral and renal stones that are not accessible by PCNL [34]. Sometimes, a ureteral access sheath or even a guidewire cannot be placed beside FUS, and thus a parallel stent for pre-stenting or an additional lithotripsy with semi-rigid ureteroscopes will be needed [15].

Some new techniques have been described to remove FUS in selective cases. Yeh et al. introduced a method using a silk loop to assist ureteroscopic lithotripsy and stent removal [35]. Mistry et al. managed mildly to moderately (< 10 mm) encrustation with insertion of a second stent next to the original stent in order to use frictional forces between the two stents causing disruption of encrustation, and then both stents were removed after 2 to 4 weeks [28].

## Conclusion

The widespread use of ureteral stents mandates updated knowledge about the management and prevention of FUS. Although FUS is uncommon, it is likely to cause troublesome and severe complications. The indications for stent insertion, especially for long-term placement, should be carefully considered in each patient. Thorough preoperative evaluation for FUS-related complications, especially the extent of encrustation, kidney function and UTIs is fundamental to developing the treatment strategy. The management of FUS should be individualized using different treatment modalities with their advantages to minimize patients’ morbidities. Patient education on timely removal of stents must be provided throughout the perioperative period. Registry and monitoring systems should be maintained for easy tracking of stents, especially in patients with poor compliance. Since the pooled data of FUS trend to be underestimated, it must be realized that it still has have a long way to go to improve the whole-process management of the ureteral stent and to strengthen the prevention of FUS.

### Electronic supplementary material

Below is the link to the electronic supplementary material.

Supplementary Material 1

## Data Availability

All data generated or analysed during this study are included in this published article [and its supplementary information files]. All data were shown in our supplementary material.
