# Supplementary material for: Forgotten ureteral stents: a systematic review of literature
Source: BMC Urol. 2024 Mar 5;24:52. doi: 10.1186/s12894-024-01440-9 (PMC10913558; doi:10.1186/s12894-024-01440-9)
Supplement: Supplementary file 1 — Supplementary Material 1 [file 12894_2024_1440_MOESM1_ESM.docx]

**Supplementary Material**

**Catalogue of included 147 literatures**

**1. Case series (in order of publication date)**

[1] Monga M, Klein E, Castaneda-Zuniga W R, et al. The Forgotten Indwelling Ureteral Stent: A Urological Dilemma. The Journal of Urology, 1995,153(6):1817-1819.

[2] BORBOROGLU P G, KANE C J. CURRENT MANAGEMENT OF SEVERELY ENCRUSTED URETERAL STENTS WITH A LARGE ASSOCIATED STONE BURDEN. The Journal of Urology, 2000,164(3, Part 1):648-650.

[3] Kehinde E O, Al-Awadi K A, Tawheed A, et al. Factors affecting the fate of prolonged forgotten 'J' stents. Scand J Urol Nephrol, 2001,35(3):222-227.

[4] Lam J S, Gupta M. Tips and tricks for the management of retained ureteral stents. J Endourol, 2002,16(10):733-741.

[5] Park K, Jeon S S, Park H, et al. Clinical features determining the fate of a long-term, indwelling, forgotten double J stents. Urol Res, 2004,32(6):416-420.

[6] YEH C, CHEN C, LIN C, et al. A New Technique for Treating Forgotten Indwelling Ureteral Stents: Silk Loop Assisted Ureterorenoscopic Lithotripsy. The Journal of Urology, 2004,171(2, Part 1):719-721.

[7] Singh V, Srinivastava A, Kapoor R, et al. Can the complicated forgotten indwelling ureteric stents be lethal?. Int Urol Nephrol, 2005,37(3):541-546.

[8] Aravantinos E, Gravas S, Karatzas A D, et al. Forgotten, encrusted ureteral stents: a challenging problem with an endourologic solution. Journal of endourology, 2006,20(12):1045.

[9] Aron M, Ansari M S, Singh I, et al. Forgotten ureteral stents causing renal failure: multimodal endourologic treatment. J Endourol, 2006,20(6):423-428.

[10] Rana A M, Sabooh A. Management strategies and results for severely encrusted retained ureteral stents. Journal of endourology, 2007,21(6):628.

[11] Acosta-Miranda A M, Milner J, Turk T M. The FECal Double-J: a simplified approach in the management of encrusted and retained ureteral stents. J Endourol, 2009,23(3):409-415.

[12] Agarwal M M, Singh S K, Mandal A K, et al. Endourologic management of forgotten encrusted ureteral stents: can ureteroscopic lithotripsy be avoided?. Surg Laparosc Endosc Percutan Tech, 2009,19(1):72-77.

[13] Eisenberg M L, Lee K L, Stoller M L. Endoscopic management of retained renal foreign bodies. Urology, 2009,73(6):1189-1194.

[14] Xu C, Tang H, Gao X, et al. Management of forgotten ureteral stents with holmium laser. Lasers Med Sci, 2009,24(2):140-143.

[15] Murthy K V R, Reddy S J, Prasad D V. Endourological management of forgotten encrusted ureteral stents. International Brazilian journal of urology, 2010,36(4):420-429.

[16] Bostanci Y, Ozden E, Atac F, et al. Single session removal of forgotten encrusted ureteral stents: combined endourological approach. Urol Res, 2012,40(5):523-529.

[17] Rabani S M. Combined percutaneous and transurethral lithotripsy for forgotten ureteral stents with giant encrustation. Nephrourol Mon, 2012,4(4):633-635.

[18] Sancaktutar A A, Adanur ^, Reşorlu B, et al. The Forgotten Ureteral Stent in Children: From Diagnosis to Treatment. The Journal of urology, 2013,189(3):1054-1060.

[19] Irkilata L, Ozgur B C, Sancaktutar A A, et al. Extracorporeal shock wave lithotripsy in the primary treatment of encrusted ureteral stents. Urolithiasis, 2015,43(4):379-384.

[20] Sohrab A, Aneesh S, Sureka S K, et al. Forgotten Reminders: an Experience with Managing 28 Forgotten Double-J Stents and Management of Related Complications. Indian journal of surgery, 2015,77(Suppl 3):1165-1171.

[21] Zahran M H, Harraz A M, Taha D, et al. Studying the Morbidity and Renal Function Outcome of Missed Internal Ureteral Stents: A Matched Pair Analysis. Journal of endourology, 2015,29(9):17-1075.

[22] Ray R P, Mahapatra R S, Mondal P P, et al. Long-term complications of JJ stent and its management: A 5 years review. Urol Ann, 2015,7(1):41-45.

[23] Adanur S, Ozkaya F. Challenges in treatment and diagnosis of forgotten/encrusted double-J ureteral stents: the largest single-center experience. Renal failure, 2016,38(6):920-926.

[24] Devraj R. Retained DJ Stent : In Terms of Morbidity and Management. International Journal of Contemporary Medical Research, 2016.

[25] Murtaza B, Alvi S. Forgotten Ureteral Stents: An Avoidable Morbidity. J Coll Physicians Surg Pak, 2016,26(3):208-212.

[26] Nerli R, Magdum P, Sharma V, et al. Forgotten/retained double J ureteric stents: A source of severe morbidity in children. African journal of paediatric surgery, 2016,13(1):32-35.

[27] Jhanwar A, Bansal A, Prakash G, et al. Endourological Management of Forgotten Double J Ureteral Stents: A Single Centre Study. SM Journal of Urology, 2017,3(1):1-3.

[28] Polat H, Yucel M O, Utangac M M, et al. Management of Forgotten Ureteral Stents: Relationship Between Indwelling Time and Required Treatment Approaches. Balkan Med J, 2017,34(4):301-307.

[29] Abdelaziz A Y, Fouda W B, Mosharafa A A, et al. Forgotten ureteral stents: Risk factors, complications and management. African journal of urology, 2018,24(1):28-33.

[30] Kartal I G, Baylan B, Gok A, et al. The Association of Encrustation and Ureteral Stent Indwelling Time in Urolithiasis and KUB Grading System. Urol J, 2018,15(6):323-328.

[31] El-Tatawy H, El-Abd A S, Gameel T A, et al. Management of 'forgotten' encrusted JJ stents using extracorporeal shockwave lithotripsy: A single-centre experience. Arab Journal of Urology, 2019,17(2):132-137.

[32] Ulker V, Celik O. Endoscopic, Single-Session Management of Encrusted, Forgotten Ureteral Stents. Medicina (Kaunas), 2019,55(3).

[33] Patil S, Raghuvanshi K, Jain D K, et al. Forgotten ureteral double-J stents and related complications: a real-world experience. African journal of urology, 2020,26(1):1-5.

[34] Vajpeyi V, Chipde S, Khan F A, et al. Forgotten double-J stent: Experience of a tertiary care center. Urol Ann, 2020,12(2):138-143.

[35] Tawfeek A M, Elmoazen M, Saafan A, et al. Simultaneous antegrade and retrograde endourological approach in Galdakao-modified supine Valdivia position for the management of missed stents associated with complex renal stones: a non-randomized pilot study. International urology and nephrology, 2021,53(2):211-217.

[36] Agrawal M, Gite V A, Sankapal P, et al. Retained ureteral stents, an avoidable source of morbidity: 10 years experience from a single tertiary care centre. Pan Afr Med J, 2022,42:68.

[37] Connelly Z M, Pilet H, Rees C D, et al. Do ureteral stent diameter and length or patient demographics play a role in stent encrustation?. Canadian Urological Association journal, 2022,16(9).

[38] Xie D, Hu C, Gu D, et al. Experiences in managing different consequences of forgotten ureteral stents. Urology annals, 2022,14(2):141-146.

**2. Case report (in order of publication date)**

[1] Schulze K A, Wettlaufer J N, Oldani G. Encrustation and stone formation: complication of indwelling ureteral stents. Urology, 1985,25(6):616-619.

[2] Persky L, Lockhart J J, Karp R, et al. The overlooked, retained Double J stent. Urology, 1990,36(6):519-521.

[3] REMBRINK K, GOEPEL M, MEYER-SCHWICKERATH M. The forgotten double J stent : case report of a multifractured ureter stent. Urologia internationalis, 1992,49(2):119-120.

[4] Fishman J R, Presto A R. The forgotten ureteral stent. West J Med, 1994,160(6):569-570.

[5] Ilker Y, Turkeri L, Dillioglugil O, et al. Spontaneous fracture of indwelling ureteral stents in patients treated with extracorporeal shock wave lithotripsy: two case reports. Int Urol Nephrol, 1996,28(1):15-19.

[6] Somers W J. Management of forgotten or retained indwelling ureteral stents. Urology (Ridgewood, N.J.), 1996,47(3):431-435.

[7] Adsan O, Guner E, Ozturk B, et al. Spontaneous fragmentation of a double J stent. Int Urol Nephrol, 1997,29(3):307-311.

[8] Mckiernan J, Katz A, Goluboff E. A long-forgotten ureteral stent. Urology (Ridgewood, N.J.), 1997,49(4):622-623.

[9] Thomas J A, Fenn N J, Ponsford A, et al. Extraction by extracorporeal shock wave lithotripsy of a forgotten ureteric stent after cystectomy. Br J Urol, 1998,81(4):643-644.

[10] Mohan-Pillai K, Keeley F J, Moussa S A, et al. Endourological management of severely encrusted ureteral stents. J Endourol, 1999,13(5):377-379.

[11] Gotwald T F, Peschel R, Frauscher F, et al. Indwelling ureteral stent fragmentation with severe encrustation and stone formation. J Urol, 1999,162(3 Pt 1):788.

[12] Sathish K S, Swami G. Urinary incontinence: an unusual manifestation of a forgotten stent. BJU Int, 1999,83(3):362.

[13] Ignjatovic I, Stojkovic I. Trapping of the double-J stent in the urinary tract eight years after extracorporeal shock wave lithotripsy. International urology and nephrology, 2000,32(1):29-31.

[14] Ivil K D, Suresh G. Incorporation of a forgotten stent into the ureteral wall. J Urol, 2001,165(6 Pt 1):1991-1992.

[15] Perera N D, Wijewardena M. Removal of severely encrusted forgotten ureteral stents by minimal access techniques. Ceylon Med J, 2002,47(1):27.

[16] Chen C, Li C, Ke H, et al. Double J Stent Forgotten For 7 Years: A Case Report. The Kaohsiung Journal of Medical Sciences, 2003,19(2):84-86.

[17] Soyupek S, Oksay T, Koşar A. Fragmentation of a forgotten double J stent and excreted with urine: case report. International urology and nephrology, 2003,35(1):91-92.

[18] Yenicesu M, Aydur E, Yildirim I, et al. A long-forgotten indwelling ureteral stent in a renal transplant patient. Transplantation Proceedings, 2004,36(5):1395-1397.

[19] Jiang J, Zhu F Q, Jiang Q, et al. Extraction of a long-forgotten ureteral stent by ureteroscopic pneumatic lithotripsy. Chin Med J (Engl), 2004,117(9):1435-1436.

[20] Madhok B, Desai R, Duttaroy D, et al. Forgotten indwelling Double-J ureteral stent: a case report and suggested treatment algorithm. . Bombay Med J, 2004(46):545.

[21] Romanowsky I, Lupu L, Lismer L, et al. Percutaneous nephrolithotomy in transplanted kidney--forgotten stent with complete staghorn and large bladder stone. Case report. Transpl Int, 2005,17(12):877-879.

[22] Polat F, Yeşil S, Kiraç M, et al. An uncommon application of shock wave lithotripsy: encrusted double pigtail ureteral stent. International urology and nephrology, 2005,37(2):231-233.

[23] Autorino R, Maschio A, Pane U, et al. The forgotten stent: late complication in a patient with neobladder. TheScientificWorld, 2006,6:410-412.

[24] Bhansali M, Patankar S, Dobhada S. Laparoscopic management of a retained heavily encrusted ureteral stent. Int J Urol, 2006,13(8):1141-1143.

[25] Eisner B, Kim H, Sacco D. Repeat knot formation in a patient with an indwelling ureteral stent. Int Braz J Urol, 2006,32(3):308-309.

[26] Lam M. Long-neglected stent in a transplanted kidney. Kidney International, 2007,71(1):5.

[27] Puhse G, Piechota H, Scheffold C, et al. Multiorgan failure 17 years after initial stone therapy: forgotten ureteral stent in a horseshoe kidney. Eur Urol, 2007,52(6):1784-1787.

[28] Whetstone J L, Smaldone M C, Gibbons E P, et al. Complete ureteral stent encrustation managed with serial nephroscopy and laser lithotripsy. Urology, 2007,69(3):515-576.

[29] Shivde S R, Joshi P, Jamkhandikar R. Extrusion of double J stent: a rare complication. Urology, 2008,71(5):814-815.

[30] Gupta R, Modi P, Rizvi J. Vanishing shaft of a double-j stent. Urol J, 2008,5(4):277-279.

[31] Ecke T H, Hallmann S, Ruttloff J. Multimodal stone therapy for two forgotten and encrusted ureteral stents: a case report. Cases journal, 2009,2(1):106.

[32] Ivica S, Dragan S. Long-term indwelling double-J stents: bulky kidney and urinary bladder calculosis, spontaneous intraperitoneal perforation of the kidney and peritonitis as a result of "forgotten" double-J stent. Vojnosanit Pregl, 2009,66(3):242-244.

[33] Lee S W, Kim J H. Renocolic fistula secondary to a perinephric abscess: a late complication of a forgotten double J stent. J Korean Med Sci, 2009,24(5):960-962.

[34] Seedat Y, Adam A. Flank pain, haematuria and poor patient compliance: beware the 'forgotten' JJ stent!. S Afr Med J, 2009,99(12):860-861.

[35] Sengottayan V K, Vasudeva P, Goel A, et al. Incontinence due to fragmentation of forgotten ureteral stent: an unusual complication. Urology, 2009,74(6):1230-1231.

[36] Tsai C, Shen J, Huang S, et al. Use of a Holmium Laser to Treat a Forgotten Double-J Stent With Whole Stent Encrustations: A Case Report. The Kaohsiung Journal of Medical Sciences, 2009,25(10):567-571.

[37] Yang P K, Agarwal D, Corcoran N. A disastrous sequela of a missed ureteric stent. Medical journal of Australia, 2009,191(10):567-568.

[38] Veltman Y, Shields J M, Ciancio G, et al. Percutaneous nephrolithotomy and cystolithalapaxy for a "forgotten" stent in a transplant kidney: case report and literature review. Clin Transplant, 2010,24(1):112-117.

[39] Ahallal Y, Khallouk A, El F M, et al. Risk factor analysis and management of ureteral double-j stent complications. Rev Urol, 2010,12(2-3):e147-e151.

[40] Davis P, Corcoran N, Cato A, et al. Images for surgeons. The forgotten ureteric stent. ANZ J Surg, 2010,80(6):453-454.

[41] Jolly E C, Adshead J M, Farrington K. The retained stent: forgotten but not gone. Kidney International, 2010,77(3):260.

[42] Niranjan A, Agarwal N, Agarwal V, et al. Enigma of forgotten double J stent. Saudi J Kidney Dis Transpl, 2010,21(1):157-159.

[43] Giridhar V, Natarajan K, Hegde P. Migration of forgotten stent into renal pelvis. Indian J Urol, 2011,27(2):282-283.

[44] Murtaza B, Niaz W A, Akmal M, et al. A rare complication of forgotten ureteral stent. Journal of the College of Physicians and Surgeons--Pakistan, 2011,21(3):190-192.

[45] Nikkhou K, Kaimakliotis H Z, Singh D. Fractured Retained Ureteral Stent in a Patient Lost to Follow-up. Journal of endourology, 2011,25(12):1829-1830.

[46] Singh D, Goel A, Ahmed N, et al. Forgotten stent leading to complex panurinary stone: single-sitting endourologic management. BMJ Case Rep, 2011,2011.

[47] Wu Z P, Zhao X K, Zhong Z H, et al. Management of the bilateral forgotten ureteral stents with open surgery. Urol Res, 2011,39(4):315-318.

[48] Kawahara T, Ishida H, Kubota Y, et al. Ureteroscopic removal of forgotten ureteral stent. BMJ Case Rep, 2012,2012.

[49] Kawahara T, Ito H, Terao H, et al. Encrusted Ureteral Stent Retrieval Using Flexible Ureteroscopy with a Ho: YAG Laser. Case Rep Med, 2012,2012:862539.

[50] Kelkar V, Patil D. Management of forgotten double J stent and severe multiple large encrusted stones in the bladder and renal pelvis. Cent European J Urol, 2012,65(4):238-241.

[51] Wani B, Upadhey R, Rathod V, et al. Forgotten long-term indwelling double "J" stent. Saudi J Kidney Dis Transpl, 2012,23(5):1043-1045.

[52] Lai D, He Y, Dai Y, et al. A long-forgotten indwelling single-J stent in a transplant kidney. Journal of the College of Physicians and Surgeons--Pakistan, 2014,24 Suppl 2:S152-S154.

[53] Wu F M W, Lim M, Deng Z, et al. Successful Endourological Management of the ‘Forgotten' Stent in a Transplanted Kidney. Urologia internationalis, 2014,92(3):373-376.

[54] Puri A, Priyadarshi V, Raizada N, et al. Forgotten DJ Stent with a Large Calculus at Its Distal End in an Ileal Conduit Diversion. Case reports in urology, 2014,2014:684651-684653.

[55] Alshumrani G. Percutaneous Antegrade Removal of Encrusted Broken Double J Ureteric Stent Using a Snare. West Indian Med J, 2014,63(5):517-520.

[56] Bardapure M, Sharma A, Hammad A. Forgotten ureteric stents in renal transplant recipients: three case reports. Saudi journal of kidney diseases and transplantation, 2014,25(1):109-112.

[57] Chaudhary R, Singh K, Dausage C, et al. Forgotten double J stents with a 'Houdini'-like vanishing act. BMJ Case Rep, 2014,2014.

[58] Isero T, Hamamoto S, Koiwa S, et al. Combined endoscopic surgery in the prone-split leg position for successful single-session removal of an encrusted ureteral stent: a case report. J Med Case Rep, 2014,8:128.

[59] Goel H K, Kundu A K, Maji T K, et al. Retained fragmented double J ureteric stent: A report of four cases with review of the literature. Saudi journal of kidney diseases and transplantation, 2015,26(4):747-750.

[60] Hikmet T, Sercan S, Ugur O H, et al. A New Method for Fragmented Ureteral Stent Extraction: Flexible Renoscopy. Urol Case Rep, 2015,3(6):190-192.

[61] Marchini G S, Torricelli F C M, Mazzucchi E, et al. Prone split-leg position to manage encrusted ureteral stents in a single-stage procedure in women: Step-by-step surgical technique. Canadian Urological Association journal, 2015,9(7-8):E494-E499.

[62] Sen V, Bozkurt H I, Yonguc T, et al. Forgotten and fragmented ureteral j stent with stone formation: combined endoscopic management. International Brazilian journal of urology, 2015,41(3):602-603.

[63] Karabıcak M, Ipekci T, Isoglu C S, et al. A rare complication after renal transplantation: Forgotten stent. Archivio italiano di urologia, andrologia, 2015,87(2):175-176.

[64] Barreiro D M, Losada J B, Montiel F C, et al. Urinary Incontinence and Urosepsis due to Forgotten Ureteral Stent. Urology Case Reports, 2016,8:63-65.

[65] Bidnur S, Huynh M, Hoag N, et al. An Indwelling Ureteral Stent Forgotten for Over 12 Years. J Endourol Case Rep, 2016,2(1):135-137.

[66] Coulier B, Lefebvre G. Forgotten Ureteral Double-J Stent Complicated by Severe Encrustation in the Bladder. Journal of the Belgian Society of Radiology, 2016,100(1):77.

[67] GU Y, ZHANG J, WANG G. Use of cystourethroscopy to remove an indwelling double-J ureteral stent 6 years following simultaneous radical sigmoid colon cancer and partial bladder resection: A case report. Experimental and therapeutic medicine, 2016,11(6):2467-2469.

[68] Talwar R, Benson M, Fam M, et al. The Open Approach to Severe Stent Encrustation: A Consecutive Case Series. Urology, 2017,99:e1-e3.

[69] Tao G, Wu G, Yang L, et al. Fragmentation of Severely Encrusted Ureteral Stent Indwelled for 4 Years in a Boy. Urol Case Rep, 2017,12:1-3.

[70] Torricelli F C M, Berjeaut R H, Laffeira L, et al. Complete Calcified Ureteral Stent: A Combined 1-Session Approach. Urology (Ridgewood, N.J.), 2017,110:259-261.

[71] Gupta R, Dey R K, Sharma R, et al. Bilateral Staghorn Calculus with Forgotten Double J Stent in Ileal Conduit Patient - A Rare Urological Challenge. J Clin Diagn Res, 2017,11(6):D9-D10.

[72] Gutierrez A, Chavarriaga J, Ocampo M A, et al. Percutaneous Nephrolithotomy, Ileal Conduit- Lithotripsy and Litholapaxy for a Neglected Encrusted Ureteral Stent. Urology Case Reports, 2017,15:17-19.

[73] Panaiyadiyan S, Shukla A, Nayak B, et al. Wandering Double-J Stent in the Retroperitoneum: A Case Report. J Endourol Case Rep, 2017,3(1):189-191.

[74] Mahmood S N, Toffeq H M, Hussen M, et al. Endourologic Management of a 15-Year-Old Neglected, Fragmented, and Encrusted Ureteral Stent. J Endourol Case Rep, 2018,4(1):201-204.

[75] Restaino S, Fanfani F, Vittori M, et al. Bilateral Ureteral Stent Removal after 15 Years: A Case Report. J Minim Invasive Gynecol, 2018,25(5):920-922.

[76] Sharma A, Garg G, Sharma D, et al. Fungal bezoar in an immunocompetent patient: a rare complication of forgotten double J stent. BMJ Case Rep, 2018,2018.

[77] Darlington D, Anitha F S. Single Session Endoscopic Removal of Bilateral Ureteric Stents Retained for Three Decades: A Case Report. Cureus, 2019,11(3):e4294.

[78] Farshid S, Sharifi-Aghdas F, Varyani M. Fragmented ureteral stent extraction by antegrade and retrograde access:using ureteroscope and nephroscope. Urol Case Rep, 2019,24:100871.

[79] Gill G S, Desai T J, Lin S Y. Encrusted Ureteral Stent in a Spanish Speaking Female: A Case of a Forgotten Stent Lost in Translation. Cureus, 2019,11(9):e5635.

[80] Kandemir A, Sonmez M G. Treatment of fragmented and severely encrusted ureteral double-J stent forgotten for 11 years through multimodal endourological methods. Urol Ann, 2019,11(3):310-313.

[81] Nesbitt A L, Joshi A, Perera M, et al. Retained neonatal ureteral stent post-ureteric reimplantation: a 26-year saga. BMJ Case Rep, 2019,12(11).

[82] Al-Hajjaj M, Kazan M N. Neglected double J stent for 8 Years with giant bladder calculi formation: A case report. Urology Case Reports, 2020,32:101195.

[83] Daly W C, Ingimarsson J P. Endourologic Management of Stent Retained Over 22 Years in Patient with Duplicated Collecting System. J Endourol Case Rep, 2020,6(4):377-379.

[84] Fuselier A, Lovin J M, Kelly E F, et al. A 22-Year-Old Retained Ureteral Stent: One of the Oldest Removed Using a Multimodal Endourologic Approach. J Endourol Case Rep, 2020,6(3):180-183.

[85] Kim D S, Lee S H. Huge encrusted ureteral stent forgotten for over 25 years: A case report. World J Clin Cases, 2020,8(23):6043-6047.

[86] Kumar S, Dutt U K, Navriya S C, et al. Ileal Ureteral Substitution After “Panureteral Damage: A Devastating Complication of Forgotten Double-J Stent”. Journal of endourology case reports, 2020,6(3):217-219.

[87] Shekar P A, Dumra A, Patel H. When the Good Old X Ray Saved the Day. Urology, 2020,141:e14-e15.

[88] Wang D, Sun H, Chen L, et al. Endoscopic combined intrarenal surgery in the prone-split leg position for successful single session removal of an encrusted ureteral stent: a case report. BMC urology, 2020,20(1):37.

[89] Zhang F, Yu J, Wang Q, et al. Urinary bladder stone due to retained indwelling ureteral stent: A case report. Medicine (Baltimore), 2020,99(39):e22293.

[90] Birowo P, Rasyid N. The use of Alken Metal Telescopic Dilator for ‘X-ray-free’ PCNL in neglected DJ stent patient: A case report. Urology Case Reports, 2020,33:101239.

[91] Aboutaleb H. A neglected double J ureteral stent for 10 years: A rare case report. Urology Case Reports, 2021,36:101570.

[92] Alam S, Ramasamy N, Thirunavukkarasu C, et al. Tubeless percutaneous nephrolithotomy (PCNL) for forgotten and retained stent in renal allograft recipient: an interesting case report and lessons learnt. BMJ Case Rep, 2021,14(1).

[93] Al-Hajjaj M, Dababo A. A missed fragmented double J ureteral stent for two years: Case report. Urology Case Reports, 2021,34:101505.

[94] Sigdel B, Shrestha S, Maskey P. Forgotten DJ stent presenting with emphysematous pyelonephritis: A life threatening complication. International Journal of Surgery Case Reports, 2021,87:106405.

[95] Ziauddin S A M, Devana S K, Sharma A, et al. Submucosal impaction of a forgotten DJ stent: addressing the unexpected. BMJ case reports, 2021,14(7):e243580.

[96] Kholis K, Palinrungi M A, Syahrir S, et al. Neglected double-J stent with giant bladder stone: a case report. Pan Afr Med J, 2021,39:213.

[97] Alwesali S M. A long forgotten ureteral stent for 13 years post renal transplantation. Urol Case Rep, 2022,44:102156.

[98] Al-Hajjaj M, Al Husein H, Kanjo M. A missed and fragmented double-j ureteral stent: A rare case report. Urology Case Reports, 2022,44:102142.

[99] Distler F A, Veelken R, Wagner A, et al. A Forgotten Ureteral Stent: Potential Risks for the Urinary Function. Urol Int, 2022,106(2):209-212.

[100] Ghorai R P, Talwar H S, Mittal A, et al. A 17-year-old indwelling ureteral stent with large vesical calculus at one end: The tombstone of a forgotten Double "J" stent. J Family Med Prim Care, 2022,11(2):796-798.

[101] Kamal W, Buksh O, Abuzenada M, et al. An endourological management of 4-year-old bilateral neglected and encrusted ureteral stents. Urol Ann, 2022,14(2):186-188.

[102] Lee I H, Shin H S, Ahn D J. A forgotten double-J ureteral stent resulting in an emphysematous perinephric abscess: A case report. Medicine (Baltimore), 2022,101(25):e29418.

[103] Mejri R, Chaker K, Bibi M, et al. Calcified double J stent removed at 10 years: a case report. Pan Afr Med J, 2022,41:94.

[104] Perez A, Nolte A C, Maurici G, et al. The "Tri-Glide" Technique: A Case Report on a Novel Intraoperative Approach for Removal of Retained and Encrusted Ureteral Stents. Case Rep Urol, 2022,2022:5708348.

[105] Tang C, Qu G, Yang G, et al. Case Report: A Calculus-Free Ureteral Stent Forgotten for 29 Years. Front Surg, 2022,9:878660.

[106] Yin S, Bai Y, Wang J, et al. Eight years of forgotten double-J ureteral stent causing damaged renal function: A case report. Asian Journal of Surgery, 2022,45(2):820-821.
